# Troponin I Levels in Neonatal Hypoxic–Ischemic Encephalopathy Are Related to Cardiopulmonary Comorbidity and Neurodevelopmental Outcomes

**DOI:** 10.3390/jcm10174010

**Published:** 2021-09-05

**Authors:** Inn-Chi Lee, Chin-Sheng Yu, Swee-Hee Wong, Ko-Huang Lue

**Affiliations:** 1 Division of Pediatric Neurology, Department of Pediatrics, Chung Shan Medical University Hospital, Taichung 402, Taiwan; a7710355@yahoo.com.tw; 2 Institute of Medicine, School of Medicine, Chung Shan Medical University, Taichung 402, Taiwan; cshy095@csh.org.tw; 3 Master’s Program in Biomedical Informatics and Biomedical Engineering, Department of Information Engineering and Computer Science, Feng Chia University, Taichung 407, Taiwan; yucs@fcu.edu.tw; 4 Department of Pediatrics, Chung Shan Medical University Hospital, Taichung 402, Taiwan

**Keywords:** newborn, HIE, biomarker, troponin I

## Abstract

Troponin I is a biomarker for cardiac injury in children. The role of troponin I in neonatal Hypoxic–Ischemic encephalopathy (HIE) may have valuable clinical implications. Troponin I levels were measured within 6 h of birth to determine their relationship to HIE stage, short-term cardiac functional outcomes, and neurodevelopmental outcomes at 1 year. Seventy-three patients were divided into two groups: mild HIE and moderate to severe HIE. Troponin I levels within 6 h of birth were obtained in 61 patients, and were significantly higher in patients with moderate to severe HIE than in patients with mild HIE (Mann–Whitney U test, U = 146, *p =* 0.001). A troponin I cut-off level of ≥60 pg/mL predicted moderate to severe HIE with a specificity of 81.1% and a negative prediction rate of 76.9%. A troponin I cut-off level of ≥180 pg/mL was significantly (χ^2^ (1, *n* = 61) = 33.1, *p* = 0.001, odds ratio 96.8) related with hypotension during first admission and significantly (χ^2^ (1, *n* = 61) = 5.3, *p* = 0.021, odds ratio 4.53) related with abnormal neurodevelopmental outcomes at 1 year. Early troponin I level may be a useful biomarker for predicting moderate to severe HIE, and initialization of hypothermia therapy.

## 1. Introduction

Birth asphyxia is a physiological disorder in newborn infants resulting from a prolonged or profound mismatch between oxygen demand and oxygen delivery [1]. Birth asphyxia is a major cause of long-term severe sequelae in children and neonatal deaths [2,3,4,5]. Birth asphyxia ranges from mild to severe. When it is moderate or severe, it can cause irreversible cerebral cell damage and death, leading to hypoxic–ischemic encephalopathy (HIE) [5,6,7]. HIE can cause disorders of multiple organs, including the brain, heart, liver, kidney, and hemopoietic system, and it may lead to an altered conscious state, autonomic instability, absence of primitive reflexes, seizures, decreased cardiac output, impairment of liver and renal function, and even death. HIE is a critical and common etiology of neonatal death and neurodevelopmental consequences [5,6].

### 1.1. Benificial Effects of Therapeutic Hypothermia Therapy

Studies have shown that therapeutic hypothermia in neonatal HIE is effective to prevent long-term neurodevelopmental consequences [7,8,9,10,11] and results in fewer effects of organic injuries in newborns [7,8,9,10]. Although therapeutic hypothermia is used clinically to reduce neurological injury secondary to HIE, there remains a 45–55% risk of death or moderate to severe disability in treated infants [7,8,10]. Therapeutic hypothermia can be used as a standard of care in HIE for moderate to severe HIE. However, hypothermia therapy is controversial in treatment of mild HIE. To date, hypothermia therapy for mild neonatal HIE has not been shown to have significant long-term benefits [11,12]. In one study, 20% of newborns with perinatal acidemia and mild encephalopathy had abnormal short-term outcomes that could be attributed to encephalopathy [13]. Murray et al. reported lower cognitive scores at 5 years in mild HIE neonates compared with controls [14]. Goswami et al. investigated the short-term outcomes of neonates with mild HIE treated with hypothermia, and exhibited that hypothermia therapy may be potentially beneficial in mild HIE of neonates [11]. The potential benefit of therapeutic hypothermia has increased pressure on clinicians to promptly and accurately assess neonatal HIE and the severity of the encephalopathy that may result [15]. Hypothermia therapy was also shown to improve function in organs other than the brain [7,8,9,10,11,16], exhibited lower mortality during first hospitalization [16], and lower hearing impairment in the hypothermia group compared with control infants [7,16].

### 1.2. Adverse Effects of Therapeutic Hypothermia

Although hypothermia is effective in moderate and severe neonatal HIE, possible side effects include the risk of intracranial hemorrhage and cardiopulmonary instability [7]. The adverse effects of hypothermia therapy include bleeding tendency, coagulation problems, and cardiovascular instability, all of which can be aggravated by HIE itself. In a meta-analysis study, 22% of 573 infants with mild HIE, underwent therapeutic hypothermia, the reported adverse effects included systemic hypotension (16%), extreme hypothermia (32%), bradycardia (100%), hypoglycemia (11%), skin necrosis (2%), thrombocytopenia (10%), coagulopathy (17%), pulmonary hypertension (6–22%), pulmonary air leak (6%) [12]. HIE-related cardiac injury during hypothermia therapy may be related to lower blood pressure and lower cardiac output, which may further increase HIE morbidity and mortality [11,12]. Fortunately, adverse events are mostly minor, and they are not necessarily associated with cooling [7]. However, Susan et al. [16] reported severe side effects during hypothermia therapy, included persistent and refractory hypotension need inotropic (46%), sepsis (6%), and overt bleeding or thrombosis (3%). Subgaleal hemorrhage (SGH) has been associated with severe hemodynamic instability, coagulopathy, and even mortality in neonatal HIE treated with hypothermia therapy [17,18]. The importance of the presence or absence of neonatal encephalopathy in predicting SGH outcomes has not been explored [17]. Although severe side effects are uncommon, hypotension and lower cardiac output during hypothermia is a critical issue, inevitably requiring the use of inotropic agents for maintaining blood pressure. Additional tools or biomarkers are required for the optimal assessment of infants with neonatal HIE in order to promptly begin hypothermia therapy if warranted.

### 1.3. Biomarkers Associated with Cardiopulmonary Comorbidity

Troponin contains three protein subunits: troponin C, troponin T, and troponin I. The heart is essentially a muscle, and damage to the heart causes it to release troponin into the bloodstream. Troponin I levels in the blood are very low (<30 pg/mL in newborns) [19], but injuries to the heart can cause the levels to increase significantly. Troponin T is a commonly used cardiac biomarker that may be useful for determining whether patients have perinatal asphyxia. Troponin T concentration was shown to be an accurate predictor of mortality before discharge [20]. Troponin I elevation is a biomarker of myocardial ischemia in adults and children. Myocardial ischemia may be a component of multiorgan injury in neonatal HIE [21]. In one study, no significant difference in troponin T concentrations was observed between cooled and noncooled neonates with moderate to severe HIE [20]. Early elevation of troponin I levels may be correlated with neonatal HIE severity and help predict residual encephalopathy in newborns at discharge from the hospital [21]. In asphyxiated term neonates, early troponin I elevation was a predictor of myocardial dysfunction, and elevated troponin T levels had high sensitivity and specificity. A cut-off troponin ≥ troponin 0.12 μg/L predicted a sensitivity of 77% and a specificity of 78% [21]. A significant direct relationship was observed between troponin values and grades of HIE [22]. Furthermore, although the relationship between troponin and the short-term and long-term outcomes of HIE may have valuable clinical implications, it has not been fully studied.

Hypothermia in newborns may induce adverse effects [11,12,23]. These conditions can cause further morbidity and mortality. Therefore, appropriate diagnosis and treatment play a critical role in the management of neonatal HIE. In this study, we investigated the comorbidities of lung and cardiac disorders in neonatal HIE treated with hypothermia therapy and the biomarkers related to the cardiopulmonary system and neurodevelopmental outcomes.

## 2. Materials and Methods

### 2.1. Patients

**Figure 1:**
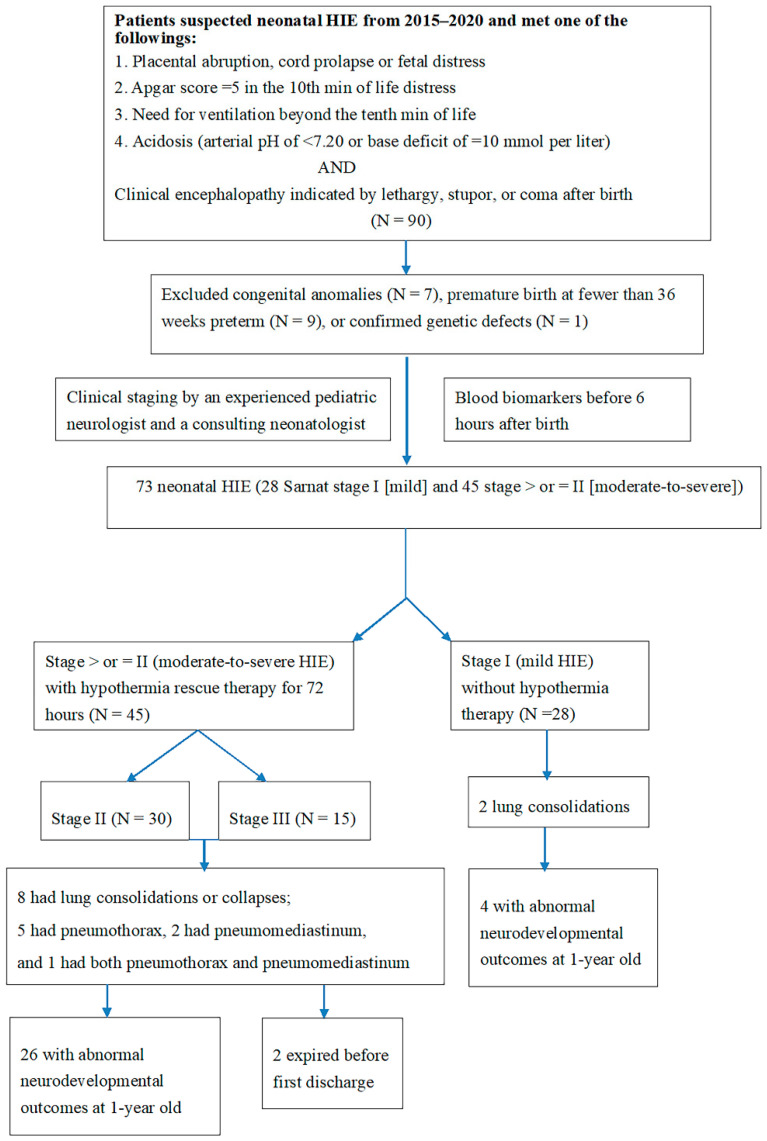
Flow chart of the study procedure. HIE indicates hypoxic–ischemic encephalopathy.

Data on neonatal HIE cases from 2015 to 2020 were collected, and the clinical history (fetal distress, metabolic acidosis, and need for positive-pressure ventilation immediately after birth) of each patient was reviewed. Ninety patients were enrolled. Seventeen patients were excluded due to congenital anomalies (*n* = 7), premature birth before 36 weeks preterm (*n* = 9), or confirmed genetic defects (*n* = 1; Figure 1). The study was conducted at Chung Shan Medical University Hospital, a medical center in central Taiwan. HIE was classified using the Sarnat staging system (stage I, mild; stage II, moderate; stage III, severe) [7,8,24].

### 2.2. Assessment of Biomarkers

Further examination of neonatal HIE included levels of troponin and creatine kinase-Mb (CKMB) by the chemiluminescence immunoassays, and creatine phosphokinase (CK). Troponin, CK, and CKMB levels within 6 h of birth were determined for neonates with HIE. Following hypothermia therapy in stage II and III HIE patients, a series of examinations were conducted before discharge, including head ultrasound (HUS) and magnetic resonance imaging. To investigate the differences in blood biomarkers for cardiopulmonary function, an experienced pediatric neurologist and a neonatologist consultant divided patients into two groups according to their Sarnat stages: mild (stage I) and moderate to severe (stage II). Differences in troponin, CK, and CKMB were compared between groups (Figure 1).

### 2.3. Investigation of Cardiopulmonary Disorders

The data of patients with parenchymal lung diseases were reviewed. Parenchymal lung diseases can be broadly divided into those that create an abnormal increase in density (lung consolidation or collapse) on a chest radiograph and those that cause increased lucency (pneumothorax or pneumomediastinum). Hypotension was defined as a mean blood pressure < gestational age in weeks [25].

### 2.4. Assessment of Neurodevelopmental Outcomes

Neurodevelopmental outcomes were evaluated at the corrected age of 1 year either through clinical evaluations conducted by an experienced pediatric neurologist or using the Bayley Scales of Infant and Toddler Development, Third Edition (Bayley-III).

Ethical approval was provided by the Chung Shan Medical University Hospital Internal Review Board (IRB #: CS2-14003), and the study was performed in accordance with relevant guidelines. Patient charts were retrospectively reviewed in all cases.

### 2.5. Statistical Analysis

Significant differences between groups were evaluated using an independent *t*-test to compare the means of two independent groups or a chi-squared test between categorical variables. The Fisher exact test was used when sample sizes was small. Logistic regression was to conduct when the dependent variable is binary. Hedges’ g (effect size) provides a measure of effect size where there are different sample sizes, and is determined by the mean difference between two groups, dividing the result by the standard deviation. The odds ratio (OR) is the association between a variable and an outcome, and is calculated by dividing the odds of the first group by the odds in the second group. If the sample distribution was nonparametric, a Mann–Whitney U test was performed. Significance was set at *p* < 0.05. All statistical tests were carried out using SPSS (version 14.0; SPSS Institute, Chicago, IL, USA).

## 3. Results

### 3.1. Demographic and Clinical Cardiopulmonary Presentations of Newborns with HIE

Overall, 73 patients had HIE after excluded 17 cases, 28 (38.4%) of 73 patients had stage I (mild) and 45 (61.6%) of 73 had stage II–III (moderate to severe) HIE receiving therapeutic hypothermia (Figure 1).

Table 1 presents the demographic data of the two groups. The birth weight, gender, age, and method of delivery were not significantly different between the two groups. However, the Apgar scores at 1 min (*t* (72) = 2.88; *p* = 0.005) and 5 min (*t* (72) = 3.62; *p* = 0.001) were significantly different between the two groups (Table 1). Fourteen (23.3%) from 73 HIE patients had parenchymal lung disorders, including 8 cases of lung consolidation or collapse among 45 patients with moderate to severe HIE and 2 cases of lung consolidation or collapse among patients with mild HIE. Pneumothorax was observed in five (11.1%) of the 45 stage II–III patients, all of whom received hypothermia therapy and endotracheal intubation. Of these five, two died, one had mild abnormal neurodevelopment, and two had normal neurodevelopment at the age of 1 year. Pneumomediastinum was observed in three patients (Table 1). One patient with isolated pneumomediastinum recovered completely.

The number of clinical cardiopulmonary presentations was higher in patients with stage II–III HIE related to hypotension during first admission (7.1% vs. 35.6%; χ^2^ (1, *n* = 73) = 7.50, *p* = 0.006,) compared with patients with stage I HIE; and higher (7.1% vs. 33.3%; χ^2^ (1, *n* = 73) = 6.63, *p* = 0.011) in parenchymal lung disorders before discharge in stage II-III HIE compared with patients with stage I HIE. 

### 3.2. Neurodevelopmental Outcomes in Patients with Mild HIE and Patients with Moderate to Severe HIE Treated with Hypothermia

Among stage I patients, four (14.3%) had mild developmental delays at the age of 1 year. In the 45 stage II–III patients treated with hypothermia therapy, two (4.4%) died before discharge due to pulmonary pneumothorax and 26 (57.8%) had abnormal neurodevelopmental outcomes at the age of 1 year (*p* < 0.001; odds ratio, 8.21; 95% CI, 2.44 to 27.60) (Table 1). Twenty-eight (62.2%) in stage II–III HIE receiving therapeutic hypothermia had abnormal neurodevelopmental outcomes or died at the age of 1 year, more (14.3% vs. 62.2%; χ^2^ (1, *n* = 73) = 16,11; *p* < 0.001) than stage I HIE with abnormal neurodevelopmental outcomes at the age of 1 year. 

### 3.3. Differences in Blood Levels of Troponin I, CK, and CKMB between Patients with Mild HIE and Moderate to Severe HIE 

**Figure 2:**
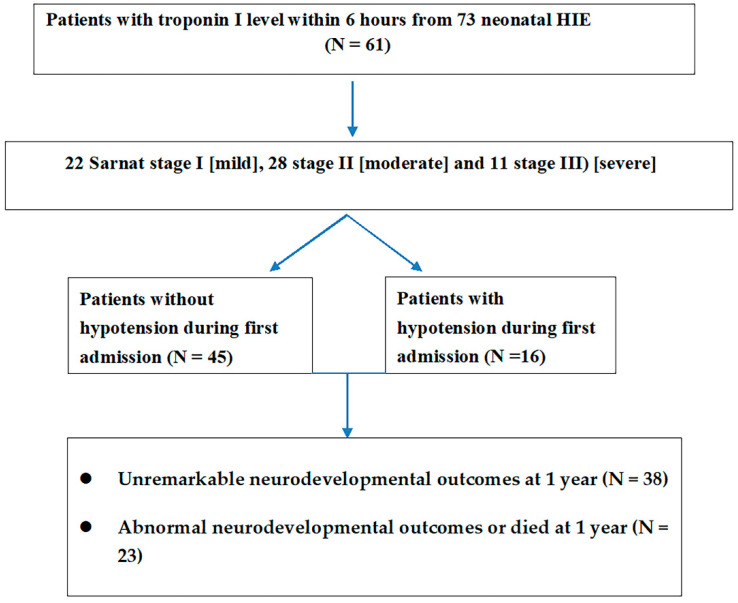
The troponin I levels were available in 61 neonatal HIE and related staging of HIE and hypotension during admission and neurodevelopmental outcomes at 1 year.

Sixty-one patients had the troponin I level within 6 hours of birth (Figure 2). Among stage I (*n* = 22) patients, the blood troponin I level was 50.6 ± 36.7 pg/mL. This was significantly lower (Mann–Whitney U test, U = 146, *p* = 0.001) than the troponin I levels of stage II–III patients (*n* = 39) (325.1 ± 754.5 pg/mL). CK and CKMB were not significantly different between the two groups. We found that troponin I levels were significantly higher in moderate to severe HIE than in mild HIE (Table 2). Troponin I levels also differed (a Mann–Whitney U test, U = 93.5, *p* < 0.001; 50.6 ± 36.7 in stage I versus 323.2 ± 764.1 in stage II) between stage I and stage II patients and between stage I and stage III (a Mann–Whitney U test, U = 52.5, *p* = 0.007; 50.6 ± 36.7 vs. 329.9 ± 768.1) patients. No difference in troponin I level was observed between patients with stage II and III HIE (Table 2). 

### 3.4. Relationship between Troponin I and Neurodevelopment Outcomes at 1 Year

Among 73 patients with HIE, troponin I data were available for 61. Of these, patients with abnormal neurodevelopmental outcomes at the age of 1 year had higher troponin I levels (607.9 ± 1158.4 pg/mL) than patients without adverse neurodevelopmental outcomes (90.3 ± 65.9 pg/mL; *p* = 0.005) (Table 3). No significant differences in troponin I, CK and CKMB were observed in blood troponin I levels between patients with and without parenchymal lung disorders (Table 3). No differences in CK and CKMB levels between patients with and without abnormal neurodevelopmental outcomes at 1 year.

A troponin I cut-off of ≥60 pg/mL was investigated to predict HIE stage, which could indicate the necessity for early hypothermia therapy (Table 4). A troponin I cut-off of ≥60 pg/mL was correlated with the staging of neonatal HIE (χ^2^ (1, *n* = 61) = 13.0, *p* = 0.001; odds ratio 7.14), with the prediction of moderate to severe HIE in a positive prediction rate (PPV) of 81.1%, a negative prediction rate (NPV) of 62.5%, a specificity of 68.2%, and a sensitivity of 76.9%. A troponin I cut-off level of ≥120 pg/mL could predict moderate to severe HIE (χ^2^ (1, *n* = 61) = 13.0, *p* = 0.005; odds ratio 9.50) with a higher PPV of 90.9% and a specificity of 90.5%, and a lower NPV of 50.0% and a lower sensitivity of 48.7% (Table 4). For patients with a troponin I level ≥60 pg/mL, this was not correlated with hypotension; however, a troponin I level ≥180 pg/mL was significantly correlated (χ^2^ (1, *n* = 61) = 33.1, *p* = 0.001; odds ratio 96.8) with hypotension during first admission (Table 4). 

**Figure 3:**
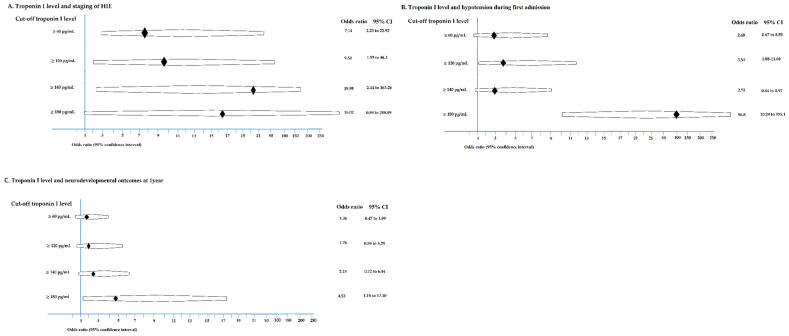
The troponin I level related to staging of HIE (**A**), hypotension during first admission (**B**), and abnormal neurodevelopmental outcome or died at 1 year (**C**) are demonstrated.

The cut-off a troponin I level can be used to correlate the neurodevelopmental outcomes at 1 year. For patients with a troponin I level ≥60 pg/mL, this was not correlated with abnormal neurodevelopmental outcomes or died at 1 year; however, a troponin I level ≥ 180 pg/mL was significantly correlated (χ^2^ (1, *n* = 61) = 33.1, *p* = 0.001; odds ratio 96.8) with abnormal neurodevelopmental outcomes or death at 1 year (χ^2^ (1, *n* = 61) = 5.3, *p* = 0.021; odds ratio 4.53) with a PPV of 66.7% and a higher specificity of 89.5%, and a NPV of 69.4% and a lower sensitivity of 34.8% (Table 4 and Figure 3).

## 4. Discussion

This study delineated the relationship between blood troponin I level and HIE stage (mild vs. moderate to severe). On the basis of these results, troponin I level can act as a blood biomarker for the early prediction of moderate to severe HIE, for which hypothermia therapy has been shown to be beneficial. This study found that CK and CKMB were not useful biomarkers of neonatal HIE staging. A troponin I cut-off of ≥120 pg/mL could be used to predict HIE stage as well as for the prediction of hypotension during first admission. However, the troponin I level ≥180 pg/mL was a biomarker for predicting neurodevelopmental outcomes in patients with neonatal HIE at 1 year.

The findings indicated that troponin I is a biomarker for cardiac cell injury and reflects the staging of HIE. One study revealed that early troponin I levels may be correlated with the severity of neonatal HIE and could predict residual encephalopathy in newborns at discharge [21]. These findings support the importance of hypotension management for HIE patients with higher troponin I levels. Taken together with this study, cardiopulmonary symptoms in neonatal HIE patients undergoing hypothermia therapy must be carefully addressed when newborns with HIE have a high troponin I level [11,12]. Appropriate transfusion of albumin or the use of inotropic agents to elevate blood pressure is suggested for patients treated with therapeutic hypothermia when the troponin I ≥120 pg/mL.

Results from previous studies on troponin I levels in neonatal HIE have been inconsistent (Supplementary Table S1) [21,22,26,27,28]. One study revealed that early troponin I levels may have been correlated with the severity of neonatal HIE and could predict residual encephalopathy in newborns at discharge [21]. Compared with our study, defining an adequate cut-off level is critical to the residual encephalopathy in neonatal HIE. In the first study, Munshi et al. [21] reported a significant difference within 6 h of age in troponin I level, but no differences at 72 h between cooled and noncooled neonates. This is concordant with our observations. Troponin T concentration was significantly higher in babies with hypotensive shock and hepatic injury but not acute kidney injury [20,28]. In one study, no significant difference in troponin T concentrations was noted between cooled and noncooled neonates with moderate to severe neonatal HIE [20]. Furthermore, troponin T levels on day 1 of life were significantly higher in babies who died than in those who survived [20].

The long-term neurological outcomes in newborns with HIE are a critical issue for clinicians. Choosing an appropriate cut-off level of troponin I is difficult and maybe biased. In this study, troponin I levels within 6 h were higher in the patients with moderate to severe HIE than in the patients with mild HIE. The troponin I levels were also higher in the patients with abnormal neurodevelopmental outcome than the level in patients with unremarkable neurodevelopmental outcomes at the age of 1 year. This was not in disagreement with the findings that the cut-off 60 pg/mL and 120 pg/mL could not predict the neurodevelopmental outcomes. An appropriate cut-off level is noteworthy. In patients treated with hypothermia therapy, neonatal hypotension could have caused secondary damage and further brain consequence. In a recent study, troponin-I levels, especially at levels >0.3 ng/mL, after therapeutic hypothermia was an independent factor for increased risk of acute kidney injury in asphyxiated newborns [29].

The proportion of abnormal neurodevelopmental outcomes was still half in moderate to severe HIE patients treated with hypothermia. This finding is comparable to those of other studies, which have demonstrated a risk of death or moderate to severe disability in 45–55% of treated infants for moderate to severe HIE [7,8,10]. Early initialization of hypothermia therapy and careful management of the critical conditions during hypothermia therapy for those neonates with moderate and severe HIE is a critical factor in long-term neurodevelopmental outcome. This study had several limitations. Data on a limited number of HIE cases were used to analyze risk factors. Therefore, studies with larger sample sizes are warranted. Another limitation could be that it is a retrospective study; therefore, our findings may be biased.

## 5. Conclusions

Troponin I levels within 6 h of birth can be a useful biomarker for determining the stage of neonatal HIE and initialize early hypothermia therapy for moderate to severe HIE. Higher troponin I ≥120 pg/mL levels were associated with hypotension during first admission. The results suggest to carefully monitor for blood pressure during hypothermia therapy. Long-term neurodevelopmental follow-up of patients is warranted. Further study to enroll a large number of neonatal mild HIE and to define an adequate cut-off troponin I level to provide early and precise management is suggested.

## Figures and Tables

**Table 1:** Demographic, clinical cardiopulmonary presentations, and neurodevelopmental outcomes of 73 newborns with HIE.

	Hypoxic–Ischemic Encephalopathy, Stage I (*n* = 28)	Hypoxic–Ischemic Encephalopathy, Stage II and III (*n* = 45)	*p* Value
Gestational age (weeks)	38.7 ± 1.2	38.6 ± 1.3	*t*(72) = 0.63, *p* = 0.723
Birth weight (gm)	3016.9 ± 331.9	2942.1 ± 413.7	*t*(72) = −0.06, *p* = 0.953
**Apgar score at one minute**	**5.4 ± 1.9**	**3.8 ± 2.3**	*t*(72) = 2.88, *p* = 0.005 *
**Apgar score at five minutes**	**7.3 ± 1.5**	**5.5 ± 2.4**	*t*(72) = 3.62, *p* = 0.001 *
Gender			χ2 (1, *n* = 73) = 0.004, *p* = 0.903
Male	17 (60.7%)	27 (61.0%)	
Female	11 (39.3%)	18 (40.0%)	
Transfer mode			χ2 (1, *n* = 73) = 0.103, *p* = 0.806
Inborn	11 (39.3%)	16 (35.6%)	
Outborn	17 (60.7%)	29 (64.4%)	
Method of delivery			χ2 (1, *n* = 73) = 0.534, *p* = 0.402
Cesarean section	13 (46.4%)	17 (37.8%)	
Vaginal delivery	15 (53.6%)	28 (62.2%)	
**Parenchymal lung disorders**	**2 (7.1%)**	**15 (33.3%) ^#^**	**χ^2^ (1, *n* = 73) = 6.63, *p* = 0.011**
Lung consolidation or collapse	2 (7.1%)	8 (17.8%)	χ2 (1, *n* = 73) = 1.65, *p* = 0.199
Pneumomediastinum	0 (0.0%)	3 (6.7%)	0.281 *
Pneumothorax	0 (0.0%)	5 (11.1%)	0.149 *
**Hypotension during first admission**	**2 (7.1%)**	**16 (35.6%)**	**χ^2^ (1, *n* = 73) = 7.50, *p* = 0.006**
Mortality before discharge	0 (0.0%)	2 (4.4%)	0.521 *
**Abnormal neurological outcomes or died at 1 year**	**4 (14.3%)**	**28 (62.2%)**	**χ^2^ (1, *n* = 73) = 16.11, *p* < 0.001**

Bold fonts indicate significance. Differences between groups were evaluated using an independent *t*-test. * In variable of Apgar score at one minute *t* (72) = 2.88, *p* = 0.005 (two-tailed hypothesis). Hedges’ *g* (effect size) was 0.74; in Apgar score at 5 min *t* (72) = 3.62, *p* = 0.001 (two-tailed hypothesis). Hedges’ *g* (effect size) was 0.85. Chi-squared test. * Fisher’s Exact Test. ^#^ One case had both pneumothorax and pneumomediastinum. HIE, hypoxic–ischemic encephalopathy.

**Table 2:** Differences in blood troponin I, CK, and CKMB levels within 6 h of birth between patients with mild HIE and patients with moderate to severe HIE.

Biomarkers	Stage I (Mean ± SD)	Stage II and Stage III (Mean ± SD)	Stage II (Mean ± SD)	Stage III (Mean ± SD)	*p* Value ^†^
**Troponin I ^±^ (*n* = 61)**	**50.6 ± 36.7 (*n* = 22)**	**325.1 ± 754.5 (*n* = 39)**			U = 146, *p* = 0.001 *
Troponin I ^±^ (stage II and stage III) (*n* = 39)			323.2 ± 764.1 (*n* = 28)	329.9 ± 768.1 (*n* = 11)	U = 134, *p* = 0.981
CK ^±^ (*n* = 61)	1701.8 ± 1813.4 (*n* = 22)	2902.4 ± 4282.3 (*n* = 39)			U = 391, *p* = 0.584
CKMB ^±^ (*n* = 36)	56.5 ± 74.7 (*n* = 15)	54.1 ± 63.8 (*n* = 21)			U = 157, *p* = 0.975
CK ^±^ (stage II and stage III) (*n* = 39)			4194.2 ± 5242.8 (*n* = 28)	1775.2 ± 2667.1 (*n* = 11)	U = 112, *p* = 0.125
CKMB ^±^ (stage II and stage III) (*n* = 21)			65.6 ± 76.4 (*n* = 13)	35.4 ± 31.0 (*n* = 8)	U = 142, *p* = 0.121

Bold fonts indicate significance. **^†^** Differences between groups were evaluated using a Mann–Whitney U test. ^*^ *Z*-Score is 4.24297. Effect size in variable of troponin I is 0.54. HIE, hypoxic–ischemic encephalopathy; ST, standard deviation; CK, creatine phosphokinase; CKMB, creatine kinase Mb. **^±^** Reference level in newborns <30 pg/mL [15]; CK reference level in newborns, 39–308 U/L; CKMB reference level in newborns, 0–4.5 ng/mL.

**Table 3:** Troponin I levels in neonatal HIEs were associated with cardiopulmonary comorbidity and neurodevelopmental outcomes at the age of 1 year.

Clinical Signs and Outcomes	Troponin I (pg/mL) (*n* = 61)	CK (U/L) (*n* = 41)	CKMB (ng/mL) (*n* = 36)
Hypotension during first admission			
Without hypotension	**250.1 ± 756.5**	2641.9 ± 3955.9	67.6 ± 76.6
With hypotension	**385.0 ± 735.9**	2367.8 ± 2737.4	38.5 ± 36.2
*p* value ^†^	U = 233, *p* = 0.037 *	U = 424, *p* = 0.804	U = 134, *p* = 0.316
Parenchymal lung disorders during first admission			
With parenchymal lung disorders	283.1 ± 710.0	1534.3 ± 1759.4	38.7 ± 53.3
Without parenchymal lung disorders	286.1 ± 764.6	2841.4 ± 3971.9	64.8 ± 71.3
*p* value ^†^	U = 292, *p* = 0.252	U = 316, *p* = 0.848	U = 107, *p* = 0.285
Neurodevelopmental outcomes at 1 year			
Unremarkable	90.3 ± 65.9	2553.9 ± 2671.9	57.9 ± 57.8
Abnormal or died	607.9 ± 1158.4	2591.9 ± 2874.8	56.5 ± 60.3
*p* value ^†^	U = 332, *p* = 0.12	U = 403, *p* = 0.093	U = 134, *p* = 0.102

Bold fonts indicate significance. **^†^** Using a Mann–Whitney U test. * Using a Mann–Whitney U test (U = 233; Z-Score is −2086). Two died before one year of age. HIE, hypoxic–ischemic encephalopathy; CK, creatine phosphokinase; CKMB, creatine kinase Mb.

**Table 4:** Outcomes associated with a troponin I cut-off level of ≥60 pg/mL and with a cut-off level ≥120 pg/mL in 61 neonatal HIE patients.

	Troponin I < Cut-Off Level (*n*)	Troponin I ≥ Cut-Off Level (*n*)	Odds Ratio (95% CI)	PPV (%)	NPV (%)	Specificity (%)	Sensitivity (%)	*p* Value ^$^ χ^2^ (df, *n*)
**A cut-off troponin I level ≥ 60 pg/mL**								
**Stage I (*n* = 22)**	**15(62.5%)**	**7 (18.9%)**	**7.14 (2.23 to 22.92)**	**81.1%**	**62.5%**	**68.2%**	**76.9%**	**χ^2^ (1, *n* = 61) = 13.0, *p* = 0.001**
**Stage II and Stage III (*n* = 39)**	**9 (37.5%)**	**30 (81.1%)**						
Patients without hypotension during first admission (*n* = 45)	20 (80.0%)	25 (20.0%)	2.40 (0.67 to 8.59)	32.4%	83.3%	44.4%	75.0%	χ^2^ (1, *n* = 61) = 1.87, *p* = 0.189
Patients with hypotension during first admission (*n* = 16)	4 (25%)	12 (75%)						
Unremarkable neurodevelopmental outcomes at 1 year ^#^ (*n* = 38)	16 (42.1%)	22 (57.9%)	1.36 (0.47 to 3.99)	40.5%	66.7	42.1%	65.2%	χ^2^ (1, *n* = 61) = 0.32, *p* = 0.495
Abnormal neurodevelopmental outcomes or died at 1 year ^#^ (*n* = 23)	8 (34.8%)	15 (65.2%)						
**A cut-off troponin I level ≥ 120 pg/mL**								
**Stage I (*n* = 22)**	**20 (90.9%)**	**2 (9.1%)**	**9.50 (1.95 to 46.3)**	**90.5%**	**50.0%**	**90.9%**	**48.7%**	χ^2^ (1, *n* = 61) = 13.0, *p* = 0.005 *
**Stage II and Stage III (*n* = 39)**	**20 (51.3%)**	**19 (48.7%)**						
**Patients without hypotension during first admission (*n* = 45)**	**33 (73.3%)**	**12 (26.7%)**	**3.54 (1.08–11.60)**	**42.9%**	**82.5%**	**73.3%**	**56.3%**	**χ^2^ (1, *n* = 61) = 4.6, *p* = 0.043**
**Patients with hypotension during first admission (*n* = 16)**	**7 (43.8%)**	**9 (56.2%)**						
Unremarkable neurodevelopmental outcomes at 1 year ^#^ (*n* = 38)	27 (71.1%)	11 (28.9%)	1.76	47.6%	67.5%	71.1%	43.5%	χ^2^ (1, *n* = 61) = 1.3, *p* = 0.25
Abnormal neurodevelopmental outcomes or died at 1 year ^#^ (*n* = 23)	13 (56.5%)	10 (43.5%)	0.59 to 5.29					
**A cut-off troponin I level ≥ 140 pg/mL**								
**Stage I (*n* = 22)**	**21 (95.5%)**	**1 (4.5%)**	**19.95 (** **2.44 to 163.26)**	**95.0%**	**51.2%**	**95.5%**	**48.7%**	**χ^2^ (1, *n* = 61) = 12.5, *p* = 0.001**
**Stage II and Stage III (*n* = 39)**	**20 (51.3%)**	**19 (48.7%)**						
**Patients without hypotension during first admission (*n* = 45)**	33 (73.3%)	12 (26.7%)	2.75 (0.84 to 8.97)	40%	80.5%	73.3%	50%	χ^2^ (1, *n* = 61) = 2.9, *p* = 0.087
**Patients with hypotension during first admission (*n* = 16)**	8 (50%)	8 (50%)						
Unremarkable neurodevelopmental outcomes at 1 year ^#^ (*n* = 38)	28 (73.7%)	10 (26.3%)	2.15 (0.72 to 6.44)	50%	68.3%	73.7%	43.5%	χ^2^ (1, *n* = 61) = 1.9, *p* = 0.166
Abnormal neurodevelopmental outcomes or died at 1 year ^#^ (*n* = 23)	13 (56.5%)	10 (43.5%)						
**A cut-off troponin I level ≥ 180 pg/mL**								
**Stage I (*n* = 22)**	**22 (100%)**	**0 (0%)**	**16.02 (0.89 to 288.09)**	**100.0%**	**43.1%**	**100.0%**	**25.6%**	***p* = 0.010 ^%^**
**Stage II and Stage III (*n* = 39)**	**29 (74.4%)**	**10 (25.6%)**						
**Patients without hypotension during first admission (*n* = 45)**	**44 (97.8%)**	**1 (2.2%)**	**96.8 (10.24 to 915.1)**	**91.7%**	**89.8%**	**97.8%**	**68.8%**	**χ^2^ (1, *n* = 61) = 33.1, *p* = 0.001**
**Patients with hypotension during first admission (*n* = 16)**	**5 (31.2%)**	**11 (68.8%)**						
**Unremarkable neurodevelopmental outcomes at 1 year ^#^ (*n* = 38)**	**34 (89.5%)**	**4 (10.5%)**	**4.53 (1.18 to 17.40)**	**66.7%**	**69.4%**	**89.5%**	**34.8%**	**χ^2^ (1, *n* = 61) = 5.3, *p* = 0.021**
**Abnormal neurodevelopmental outcomes or died at 1 year ^#^ (*n* = 23)**	**15 (65.2%)**	**8 (34.8%)**						

Bold fonts indicate significance; df, degrees of freedom; **^$^** chi-squared test. **^%^** Fisher’s exact test. HIE, hypoxic–ischemic encephalopathy; PPV, positive prediction rate; NPV, negative prediction rate. CI indicates confidence interval. **^#^** Two case died before first discharges.

## Data Availability

The datasets used and/or analyzed during the current study are available from the corresponding author on reasonable request.

## Supplementary Materials

The following are available online at https://www.mdpi.com/article/10.3390/jcm10174010/s1, Table S1: The literature review for troponin level correlated with neonatal HIE.
