# Supplementary material for: Troponin I Levels in Neonatal Hypoxic–Ischemic Encephalopathy Are Related to Cardiopulmonary Comorbidity and Neurodevelopmental Outcomes
Source: J Clin Med. 2021 Sep 5;10(17):4010. doi: 10.3390/jcm10174010 (PMC8432483; doi:10.3390/jcm10174010)
Supplement: Supplementary file 1 [file jcm-10-04010-s001.zip › jcm-1355214-supplementary.pdf]

Supplementary Table S1. The literature review for troponin level correlated with neonatal HIE.

Authors	Methods and patients	Results	Significances	Reference
Munshi et al., 2020	104 newborns with hypothermia for HIEs	Significant elevation of troponin I in mild-to-moderate HIE and severe HIE as compared with control	A cut-off troponin ≥ troponin 0.12 μg/L predicted a sensitivity 77%; specificity 78% ; PPV 68%; NPV 84% for residual encephalopathy	[21]
Alkholy et al.,2017	25 HIEs and 25 healthy control	Cord CKMB at 12.5 U/L had 100% sensitivity and 84% specificity in the detection of severe HIE infants	Serum CKMB in HIE infants were significantly increased early in HIE.	[26]
Shastri et al., 2012	60 neonatal HIEs	Serum troponin I and duration of inotropic support were significantly greater with increasing severity	Troponin before 36 hours of birth correlate strongly with HIE grading and with duration of inotropic use.	[27]
Bhasin et al., 2019	23 neonatal stage I, 10 stage II and 8 stage III HIEs, and with non-HIEs (control)	Troponin T raised in 13 (56.5%) HIE. CK, CKMB and troponin were higher (p < 0.05) in non-survivors.	Cardiac enzymes changes associated with increasing severity and mortality.	[28]
Joseph et al., 2018	120 neonatal HIEs	Myocardial dysfunction had higher troponin T (0.277±0.231 ng/ mL) than without myocardial dysfunction (0.061±0.036 ng/ mL, p < 0.005).	Significant relation with increasing troponin values and increasing grading of HIE	[22]

HIE indicates hypoxic-ischemic encephalopathy; PPV, positive predictive value; NPV, negative predictive value; CK, creatine phosphokinase; CKMB, creatine kinase Mb.
